# High school students' social media use predicts school engagement and burnout: the moderating role of social media self-control

**DOI:** 10.3389/frcha.2024.1269606

**Published:** 2024-08-13

**Authors:** Jie Du, Yu Wang

**Affiliations:** ^1^ Research Center for Digital Intelligence Strategy and Talent Development, Chongqing Technology and Business University, Chongqing, China; ^2^ Research Center for Enterprise Management, Chongqing Technology and Business University, Chongqing, China; ^3^ School of Business Administration, Chongqing Technology and Business University, Chongqing, China; ^4^ Management School, Chongqing University of Technology, Chongqing, China

**Keywords:** social media, self-control, high school students, engagement, burnout

## Abstract

Students' social media use has quickly gained attention given the effect of considerable time spent on and widespread usage of social media on their development and success. The study aimed to examine whether high school students' social media use predicts more school engagement and less burnout for those who were more successful in controlling their social media use in goal-conflict situations. A sample of 107 Chinese high school students (*M*_age_ = 19.21, *SD*_age_ = 1.85, 68% female) participated in an online survey. The results showed that social media self-control failure moderated the relationship between general social media use (rather than social media use intensity) and school engagement. A simple effect test revealed that more general social media use predicted higher school engagement for students who were more successful in controlling their social media use. However, no moderation effect was observed of social media self-control failure on the relationship between social media use intensity (or general social media use) and burnout. The results partially supported the study demands-resources model and indicated the potential benefits of controllable social media use on high school students’ engagement in the face of high academic demands.

## 1 Introduction

School engagement and burnout have been a significant research focus for educators and researchers for many years because they reflect the overall academic and psychological functioning of students (1). The demands-resources model has been widely used to examine the predictors of student engagement and burnout (2–5). Following this model, a number of studies on adolescents' engagement and burnout have been increasing to improve students' motivation toward study and to increase successful student achievement levels (3, 6). However, only a few studies have focused on the possible role of social media use.

Students' social media use has quickly gained attention given the effect of considerable time spent on and widespread usage of social media on their development and success (7, 8). Chinese high school students are known to have a high study load and academic demands in the face of the competitive National College Entrance Examination (5). This is particularly true for students in senior grades and return students (students who reattend classes after failing the college entrance examination). Social media use is popular in Chinese students, as its use can help to release pressure and fulfill several important gratifications of students (e.g., stay connected to social networks, organize and participate in relevant events and feel involved on campus) (9).

However, when using social media disturbs other school-related goals and tasks, many students fail to resist the temptation of social media use, which is also called “social media self-control failure” (10). Different from more extreme problematic (e.g., addictive or compulsive) use and more general dysfunction of self-control, social media self-control failure describes a momentary and intermittent self-control problem that many users experience daily (10). However, it was found to relate to negative consequences such as postponing school-related work and generating negative emotions (e.g., time pressure, guilt and academic strain) (10–12). The pros and cons of students' social media use stimulate a growing practitioner and academic interest in understanding its role in students' school performance and wellbeing. The main focus of the current literature has been on the maladaptive use of social media, such as addictive social media use (13). However, the positive impact of social media use on high school students' mental health and study performance is relatively ignored, taking into account self-control as an important boundary between social media use and the outcome measures.

We proposed that the balance of the social media effect depends on whether students can successfully control their social media behaviors when it conflicts with other goals. In other words, self-control may serve as a moderator that determines the effects of social media use on students' engagement and burnout. In addition to several review studies in media research that underlie the moderating role of self-control (14–16), no study has empirically examined this effect in high school students. Thus, using the demands-resources model as a guiding framework, the present study aimed to answer the question of whether social media-related self-control moderates the prediction of high school students' social media use on engagement and burnout.

### 1.1 Student engagement and burnout

The definition of engagement in the educational context varies by coverage and emphasis in different studies (17, 18). Generally, scholars have concluded that student engagement is a positive state of vigor, dedication and absorption toward school and study-related activities (19–21). Engagement has a positive impact on several important aspects of school life, such as GPA (22) and life satisfaction (23).

Engagement focuses on students' mindset of motivation, which is considered a multidimensional construct (21). One often-used multidimensional model of student engagement is characterized by three facets that reflect psychological involvement in study-related activities: vigor, absorption and dedication (19, 21). Vigor refers to the mental aliveness and behavioral endeavor to school activities and the persistence toward study tasks. Dedication reflects the aspect of identification in school, including inspiration, pride, and enthusiasm in academic learning; Absorption describes the deep concentration in tasks and activities of study (21). Based on the three-component model of engagement, Martin (19) developed a self-report engagement scale, which was validated in 12,237 high school students from 38 Australian high schools. Following this research, a Chinese version of the student engagement scale was validated in 2,330 high school students and showed excellent psychometric properties (24).

Student burnout refers to a state of mental and physical exhaustion toward school and the perception of inadequacy as a student (21). Burnout describes an extinction of motivation or incentive from unwanted results. It is reflected as deficient energetic resources and low dedication toward study (25). Burnout was found to predict poorer academic achievements in a meta-analysis of 29 studies (26). Moreover, it was also an indicator of school drop-out rate even from the data of Finland, known for equality-striving and high-quality educational system (27).

The components of burnout are commonly concluded to be three constructs that reflect the opposite attribute of engagement: emotional exhaustion, lack of personal accomplishment and cynicism (20). Emotional exhaustion describes the perception of weariness and fatigue toward study. Students suffering from burnout were chronically drained from their school tasks and devoted less energy to study. Reduced personal accomplishment refers to the loss of competence and achievement at school. Students often feel inadequate to be a student or unable to reach their study goals. Cynicism refers to a distant attitude toward study. Cynical students often express their hatred or indifference toward study (20). Based on the three components of student burnout, Wu et al. (28) established a Chinese version of the Student Burnout Inventory and validated its psychometric properties in 3,386 students from primary school to high school (28).

### 1.2 Demands-resources model and high school students' social media use

The demands-resources model provides a theoretical lens for understanding the effects of students' social media use on engagement and burnout (20). The demands-resources model originated from the job demands-resources model, which has been well used to explain occupational engagement and burnout (29). Based on the job demands-resources model and empirical evidence from educational studies, Salmela-Aro et al. (20) developed a study demands-resources model to explain the antecedents and development process of student engagement and burnout.

This model distinguishes two parallel but opposite factors that lead to school engagement and burnout: resources and demands. Resources are the physical, mental, and social aspects of study that help students achieve their study goals, reduce study demands or stimulate their personal growth (20). A typical resource is the feedback of students' performance obtained from school, which helps to evaluate the gap between current status and academic goals (2). Another core resource is emotional support from teachers (30) and significant others (31). Moreover, resources include students' personality factors that relate to self-autonomy. For instance, self-efficacy (i.e., feelings of competence in study due to the achievement of good results) was demonstrated to promote high school students' engagement (32). Self-control was also found to positively predict all three dimensions of engagement in undergraduate students (33).

In an early review, Heiberger & Harper (9) discussed the potential of Facebook use as a resource to increase college students' engagement. Based on the evaluation of practices in educational settings, they argued that Facebook is a useful tool for increasing social integration and the climate of commitment in college students. Facebook can be used for social interaction between students, faculty and staff members. It was also an effective application for programming initiatives, as well as information exchange for campus student issues (9). Overall, the use of Facebook was considered an opportunity and resource in promoting student involvement and engagement. Some empirical findings also supported the linkage between social media use and engagement. For instance, Mazer et al. (34) found that teachers' self-disclosure on Facebook was linked to higher levels of motivation in college students (34). Another investigation among undergraduate students' Facebook use and their engagement further revealed that only some Facebook activities (e.g., RSVP to events and viewing photos) were positively related to engagement. Other activities, such as playing games, posting photos, and Facebook chatting, were negatively related to engagement (35). The findings stress the necessity of distinguishing general social media usage and usage of different social media activities in predicting engagement.

Demand factors include physical, mental and organizational pressure that generate stress for students (20). According to the demands-recourses model, study demands, such as too much school work and too many courses, are the core indicators of burnout because these factors could generate mental and emotional burdens (20). When students experience depletion of energy without gaining sufficient returns, they are more likely to feel fatigue and exhaustion (4). Nevertheless, there are individual differences in perceiving the objective nature of the study environment. For example, when confronted with equal study load, some students perceive less stress, while others are easily overwhelmed. This is because individual factors such as autonomy and locus of self-control were identified to cope with stressors generated from demands (2).

Theoretically, social media can be used as a tool to help students recover from academic stress [e.g., (36)], thus preventing students from burnout. However, previous studies have shown that social media use is also a main source of distraction (37). Social media was found to often distract people from study, work, sports, reading, sleep, family time, and house duties (38, 39). This makes social media a possible risk factor for burnout. For high school students faced with high study demands, social media is an efficient tool for finding support from family and friends (40) and becoming entertained after study. However, this could motivate more social media usage and make students more easily distracted from pursuing other important goals (37). A study found that social media disrupted students from study-related activities, which created more time pressure (41). Another study found that social media use postponed school-related work, which may elevate students' academic stress (11). The evidence suggests that social media use could intensify study demands, which further predicts burnout.

### 1.3 The moderating role of social media self-control

As stated above, social media use can facilitate connection to study resources that will promote engagement and aggravate study demands that will lead to burnout. Thus, it is necessary to clarify the conditions under which social media use will help obtain resources and will create more study demands. We assumed that self-control plays an important role in leveraging this process. The literature on media use has identified self-control as an important moderator in the relationship between social media use and healthy outcomes such as wellbeing (14). It was argued that when social media use conflicts with other important goals (e.g., getting entertainment vs. reaching study goals), people need to exert self-control to regulate their social media behaviors (16, 36). Self-control prioritizes more important goals over short-term media pleasure by eliciting negative emotions regarding social media use (e.g., guilt, time pressure, academic strain) (11) and inhibiting the impulsive enactment of social media behaviors (42). From the perspective of the demands-resources model, successful control over social media use can benefit study resources through more conscious use of social media in favor of academic goals. This can enhance students' efficacy toward study, such as actively using social media to obtain feedback from teachers and peers (43) and conducting distance learning (6), which promotes engagement.

In contrast, a lack of self-control over social media can increase the risk of burnout. This is because social media use may aggravate the stress and workload from study demands by creating more goal conflicts between excessive social media use and study goals (11). This also supports the idea regarding motivational interference. According to this theoretical framework, students often face conflicts between their motivation to learn and other alternative activities (e.g., talking to friends or watching TV). When the alternative activity is more emotional rewarding than concurrent learning goal, it can interfere with the ability to regulate one's learning (44, 45). Similarly, social media use can be also seen as an attractive alternative activity that disrupts self-control in studying. Students with an impaired self-control are more likely to be drawn to social media activities that offer high emotional rewards. For instance, they may spend with more time browsing social networking sites or watching short videos instead of finishing school-related work. Leaving goals and tasks unfinished will cause competitive pressure (46) and create more strain toward studying (11), increasing the risk of burnout.

Evidence from empirical studies supported the moderating role of self-control between social media use and engagement and burnout. For instance, a study surveyed 210 social media users and found that a lack of impulse control in checking notifications on social media predicts more feelings of fatigue over social media use (47). A study among 634 college students found that using media during class predicted more burnout, particularly through a deficient level of self-control (48). An experimental study compared two groups of students who were allowed or not allowed to use Facebook messaging during class. The results showed that being unable to regulate social media multitasking reduced students' academic performance in class (49).

**Figure 1:**
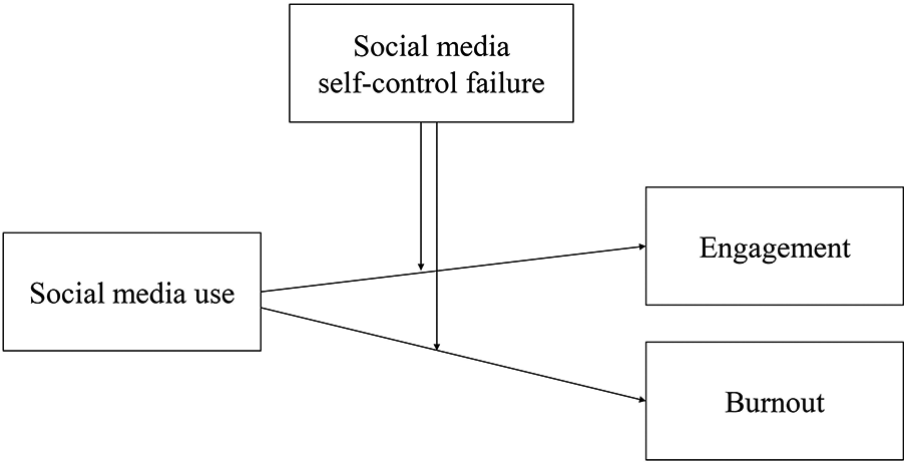
Social media self-control failure moderates the relationship between social media use and school engagement and burnout.

Taken together, being able to employ self-control to resist the temptation of social media in goal-conflict situations may serve as a moderator that alters the effect of students' social media use on engagement and burnout. Based on the demands-resources model and the above empirical evidence, we hypothesized that high school students' social media use will predict higher school engagement and lower burnout for those who are more successful (vs. less successful) in controlling their social media use in goal-conflict situations (see Figure 1). In addition to the independent variable and moderator, several control variables were also considered: self-control, depletion sensitivity, general social media use, and academic pressure. Self-control refers to a more general ability of impulse inhibition (50), while depletion sensitivity reflects the individual differences regarding the ratio of exhaustion under high cognitive load (51). The two measurements were found to be highly related to social media-induced self-control failure (10). We considered that the two constructs reflected a general ability students may exhibit in their social media use and study. Compared with self-reported educational status (e.g., exam scores) which could be biased due to inaccurate memory and social desirability, the measures could better reflect a trait-like aspect of individual differences in their school performance. Therefore, we estimated the moderation effect while controlling for the effects of general self-control capacity and depletion sensitivity. Academic pressure represents the subjective perception of study demands, and general social media use represents the time spent on and frequency of visit to social media. These two measures might vary among different high school students (43). To eliminate personal factors that may confuse the results, we controlled the above variables while testing the hypothesis.

## 2 Method

The study was approved for exemption by the research ethics committee of the first author's university. Before data collection, we preregistered the research question, hypotheses, sample size, main variables to be collected and data exclusive criteria (see the preregistration file at https://aspredicted.org/wm2re.pdf). The original data and analytical script were uploaded to Open Science Framework (https://osf.io/kf783/).

### 2.1 Participants and procedure

Based on power analysis with a median effect (0.15) of the *F*-test, we aimed to collect 103 Chinese high school students. Participants were recruited through an online participant pool Credamo (https://www.credamo.com). Participants first provided consent by answering whether they were currently studying in high school and used social media and their agreement on participation. Then, they were instructed to answer questions about their social media use intensity, social media self-control failure, engagement, burnout and control variables (i.e., self-control, depletion sensitivity and academic pressure). Then, they provided demographic information. Each participant was paid 5 CNY for their participation.

The initial sample includes 110 participants. Following the preregistration, participants were excluded if they (1) did not use social media, (2) did not agree to participate, (3) completed the investigation faster than ±3*SD* of the average completion time, (4) had missing values in the questionnaire, and (5) were identified as duplicated cases. The final sample included 107 participants aged between 18 and 25 (*M* = 19.21, *SD* = 1.85), and 68% were female. Participants were mostly from the 3rd grade of senior high school (45%) and were mostly from the central districts of China (47%). The most commonly used social media was WeChat (96%) (Table 1).

**Table 1:** Demographic information and most commonly used social media platforms.

Grade		Most commonly used social media platform^a^	
1st grade	2 (2%)	Wechat	103 (96%)
2nd grade	11 (10%)	Sina Weibo	37 (35%)
3rd grade	48 (45%)	QQ	95 (89%)
Return student	46 (43%)	Bilibili	57 (53%)
Location of residence		TikTok	84 (79%)
Eastern district	28 (26%)	Redbook	60 (56%)
Central district	50 (47%)	QQ zone	28 (26%)
Western district	26 (24%)	Baidu Tieba	3 (3%)
Northwestern district	3 (3%)	Meipai	2 (2%)
		Douyin Huoshan	5 (5%)
		Other	4 (4%)

^a^ The options of the mostly used social media platforms were based on the investigation by Simon (52).

### 2.2 Measurements

Social media use was measured by the 14-item Social Networking Activity Intensity Scale (SNAIS) (53). The SNAIS includes two subdimensions: social function use intensity and entertainment function use intensity, both of which reflect the leisure use of social media (i.e., keeping in contact with old friends, entertainment use and making new friends) in Chinese adolescents. Participants were asked: “How often have you performed the following online social networking activities in the last month?” Participants rated each of the social media activities (e.g., “Sent messages to friends on message board”, “Surfed entertainment/current news”) on a 6-point Likert scale (0 = never, 1 = few, 2 = occasional, 3 = sometimes, 4 = often, 5 = always). An overall average score including both dimensions was computed. A higher score indicates higher social networking use intensity. Confirmative factor analysis showed a 2-dimensional structure of the scale [χ^2^ = 80.3, *df* = 71, *p* = .210, CFI = 0.97, TLI = 0.96, SRMR = 0.07, RSMEA = 0.04, 90% CI (0.00, 0.07)].

School engagement was measured by the Utrecht Learning Engagement Scale (UWES) (24). The revised UWES includes 9 items (e.g., “When I am studying, I forget everything else around me” and “I can continue for a very long time when I am studying”). Participants were asked to rate each item according to their agreement with the statement on a 7-point scale, 1 = never, 2 = rarely (once or twice a year and less), 3 = occasionally (once or twice a month and less), 4 = sometimes (2–4 times a month), 5 = often (about once a week), 6 = very often (2–4 times a week and more), 7 = always (about once a day). An average score was computed to represent high school students’ school engagement. A higher score indicates higher school engagement. The scale showed a unidimensional structure [χ^2^ = 35.69, *df* = 25, *p* = .076, CFI = 0.97, TLI = 0.96, SRMR = 0.04, RSMEA = 0.06, 90% CI (0.00, 0.11)].

Burnout was measured with the 16-item Chinese Adolescent Student Burnout Inventory (28). The scale includes three dimensions: physical and mental exhaustion, study alienation and low accomplishment. The physical and mental exhaustion subscale measures exhaustion and tiredness due to study (“After a day of study, I felt very tired”). The study alienation subscale measures the passive attitude toward study (“I think that study is meaningless to me”). The low accomplishment subscale measures the diminished personal accomplishment regarding study (“I can often achieve my goals”, reverse scored). An averaged score was used to represent student burnout. A higher score indicates a higher tendency of burnout. The original 3-dimensional structure was confirmed in the present study [χ^2^ = 125.06, *df* = 97, *p* < .05, CFI = 0.96, TLI = 0.95, SRMR = 0.07, RSMEA = 0.05, 90% CI (0.02, 0.08)].

Social media self-control failure was measured with the Social Media Self-Control Failure (SMSCF) scale (10). The Chinese version of the scale was validated in a sample of 2,400 university students (54). The scale includes 3 items measuring the extent to which people fail to control their social media use in goal-conflict situations. Participants were asked “How often do you give in to a desire to use social media even though your social media use at that particular moment: (1)…conflicts with other goals (for example: doing things for school/study/work or other tasks)? (2)…makes you use your time less efficiently? (3)…makes you delay other things you want or need to do?” Each item was rated on a 5-point scale (1 = Never, 5 = Always). An average score was computed to represent the extent of social media self-control failure. A higher score indicates a higher tendency to fail to control one's social media use in goal-conflict situations. Principal component analysis with oblimin rotation showed that the scale had a unidimensional structure with an eigenvalue of 2.18. The three items explained 72.8% of the variance.

General social media use was measured with four questions about the frequency of visits to social media and time spent on social media (37). As smartphone use is not allowed in some Chinese high schools during weekdays, participants reported their general social media use on weekdays and weekends: (1) “During weekdays/weekends, approximately how many minutes per day do you spend on social media?” (1 = 10 min or less, 2 = 11–30 min, 3 = 31–60 min, 4 = 1–2 h, 5 = 2–3 h, 6 = more than 3 h), (2) “During weekdays/weekends, how often do you visit social media?” (1 = Less than once a day, 2 = Once a day, 3 = 2–3 times a day, 4 = once an hour, 5 = 2–3 times an hour, 6 = More than 3 times an hour). Spearman's ρ between the four items was 0.33–0.46, all *ps* < .001. We averaged the four items to represent overall social media use.

Self-control was measured by the 7-item Brief Self-Control Scale (BSCS) (55). The Chinese version of the scale was validated in a sample of 1,676 university students (56). Participants were asked to rate each item (“I am good at resisting temptations”, “I do certain things that are bad for me if they are fun”) on a 5-point Likert scale from 1 (not at all) to 5 (very much). An average score was computed. A higher score indicates a higher level of self-control capacity. The original 2-dimensional structure of the scale was confirmed in the present study [χ^2^ = 11.06, *df* = 12, *p* = .524, CFI = 1.00, TLI = 1.01, SRMR = 0.04, RSMEA = 0.00, 90% CI (0.00, 0.09)].

Academic pressure was measured by the academic pressure subscale from the Mental Health Inventory of Middle-school students (57). The subscale includes 6 items measuring middle school students' perceived pressure related to the burden of study, fear of being questioned by teachers, aversion to homework, being nervous about exams and so forth (e.g., “I felt a heavy burden of study”, “I hate doing homework”). Participants rated each item on a 5-point scale (1 = never, 2 = slight, 3 = moderate, 4 = moderately severe, 5 = severe). An average score was computed. A higher score indicates a higher level of academic pressure. The original unidimensional structure of the scale was confirmed in the present study [χ^2^ = 15.68, *df* = 9, *p* = .074, CFI = 0.97, TLI = 0.96, SRMR = 0.04, RSMEA = 0.08, 90% CI (0.00, 0.15)].

Depletion sensitivity was measured by the 11-item Depletion Sensitivity Scale [DSS; (51)]. The scale assessed individual differences regarding how easily one's self-control resource is drained after a self-control demanding task (“After I have made a couple of difficult decisions, I will be mentally fatigued”, “I get mentally fatigued easily”). Participants rated each item on a 7-point scale ranging from 1 (totally disagree) to 7 (totally agree). An average score was computed. A higher score indicates a higher level of depletion sensitivity. The original unidimensional structure of the scale was confirmed in the present study [χ^2^ = 57.80, *df* = 43, *p* = .065, CFI = 0.96, TLI = 0.95, SRMR = 0.05, RMSEA = 0.06, 90% CI (0.00, 0.09)].

### 2.3 Analytical strategy

Data analysis was conducted with jamovi (version 2.3) (58). We first conducted descriptive analysis of the key variables of the model. Then moderation analysis was conducted using bootstrap estimation (sample = 1,000). Using hierarchical multiple regression analysis, we further analyzed the moderation effect while controlling for the influence of several control variables based on their associations with the dependent variables. Moreover, gender was included in the model test as a control variable when examining the prediction of social media use to burnout, because female participants showed higher level of burnout than male participants (*t* = −2.37, *df* = 105, *p* = 0.020).

## 3 Results

### 3.1 Descriptive results

The descriptive statistics and correlation matrix of the main variables and control variables are presented in Table 2.

**Table 2:** Descriptive statistics and correlation matrix of the variables.

	1	2	3	4	5	6	7	8	9	10
1. SMUI	–									
2. SE	0.18	–								
3. Burnout	−0.03	−0.65***	–							
4. SMSCF	0.07	−0.51***	0.60***	–						
5. SMU	0.23*	−0.17	0.22*	0.25*	–					
6. AP	−0.01	−0.51***	0.73***	0.48***	0.14	–				
7. SC	0.06	0.63***	−0.63***	−0.58***	−0.15	−0.54***	–			
8. DS	0.01	−0.56***	0.64***	0.50***	0.15	0.61***	−0.71***	–		
9. Age	0.00	0.07	−0.11	0.03	0.28**	−0.08	0.08	0.05	–	
10. Grade	−0.05	0.20*	−0.16	−0.18	0.11	−0.16	0.08	−0.03	0.46***	–
*M*	3.78	3.87	2.65	3.33	4.05	2.98	2.93	4.69	19.21	3.29
*SD*	0.64	0.93	0.70	0.80	1.08	0.85	0.78	0.99	1.85	0.73
*Max*	1.29	1.67	1.25	1.00	1.75	1.33	1.14	1.73	16	–
*Min*	5.21	6.22	4.81	5.00	6.00	4.67	4.57	6.64	25	–
ω	0.80	0.87	0.88	0.81	–	0.86	0.84	0.87	–	–

SMUI, social media use intensity; SE, study engagement; SMSCF, social media self-control failure; SMU, social media use; AP, academic pressure; SC, self-control; DS, depletion sensitivity.

* *p* < .05.

** *p* < .01.

*** *p* < .001.

### 3.2 The moderating role of social media self-control failure on the relationship between social media use intensity and engagement

The results of moderation analysis showed no moderation effect of social media self-control failure on the relationship between social media use intensity and school engagement (*b* = 0.11, *Z* = 0.63, *p* = .527, 90% CI [−0.37, 0.31). However, the main effect of social media intensity and social media self-control failure was observed. Overall, students who had a higher intensity of social media use also showed higher school engagement [*b* = 0.34, *Z* = 2.74, *p* < .01, 90% CI (0.15, 0.63)]. Students who often fail to control their social media use in goal-conflict situations showed less school engagement [*b* = −0.59, *Z* = −5.76, *p* < .001, 90% CI (−0.78, −0.38)].

After controlling for general self-control capacity, depletion sensitivity, general social media use, age and grade, no interaction was found between social media use intensity and social media self-control failure. Neither a main effect of social media use intensity nor social media self-control failure was observed (Table 3).

**Table 3:** Moderation effects of social media use intensity on the relationship between social media self-control failure and the outcome measures.

Variables	Engagement				Burnout			
	Model 1		Model 2		Model 1		Model 2	
Control variables	*b*	*SE*	*b*	*SE*	*b*	*SE*	*b*	*SE*
Academic pressure	−0.18	1.00	−0.13	0.10	0.38***	0.06	0.35***	0.06
Self-control	0.49***	0.10	0.31*	0.14	−0.20*	0.08	−0.11	0.09
Depletion Sensitivity	−0.15	0.13	−0.18	0.11	0.12	0.07	0.12	0.07
General social media use	−0.07	0.11	−0.11	0.07	0.08	0.04	0.07	0.04
Age	−0.01	0.07	0.02	0.04	−0.03	0.03	−0.04	0.03
Grade	0.20	0.04	0.14	0.11	−0.03	0.07	0.01	0.07
Gender					0.10	0.10	0.05	0.10
Social media use intensity (SMUI)			0.20	0.32			−0.13	0.20
Social media self-control failure (SMSCF)			−0.14	0.39			0.11	0.25
SMUI × SMSCF			0.01	0.10			0.02	0.06
*F*	14.92***	11.53***			25.23***	19.40***		
*R^2^*	0.47	0.52			0.64	0.67		
Δ*R^2^*		0.04*			0.03*			

Coefficients were standardized.

* *p* < .05.

*** *p* < .001.

### 3.3 The moderating role of social media self-control failure on the relationship between social media use intensity and burnout

No moderation effect of social media self-control failure was found on the relationship between social media use intensity and student burnout [*b* = −0.00, *Z* = −0.02, *p* = .981, 90% CI (−0.26, 0.08)]. However, the main effect of social media self-control failure was observed. Overall, students who often failed to control their social media use in goal-conflict situations showed more burnout [*b* = 0.53, *Z* = 7.07, *p* < .001, 90% CI (0.38, 0.69)]. However, after controlling for academic pressure, general self-control capacity, depletion sensitivity, general social media use, age, gender and grade, the hierarchical multiple regression analysis showed no interaction or main effect of social media use intensity and social media self-control failure on burnout (Table 3).

### 3.4 Exploratory analysis

We further analyzed the moderation of social media self-control failure on the relationship between general social media use and the outcome measures. This is because social media intensity describes the *intensity* of use in different social media activities. It would also be interesting to explore whether the general *usage* of social media could predict the two outcome measures at different levels of social media self-control failure. Thus, we used hierarchical multiple regression to examine whether general social media use predicts school engagement and burnout for students who had different levels of social media self-control failure while controlling for academic pressure, self-control, depletion sensitivity, social media use intensity, age and grade.

Regarding school engagement, the results showed an interaction between general social media use and social media self-control failure (Table 4). A simple effect test showed that a higher level of general social media use predicted more engagement when the prediction was estimated at a lower level (−1 *SD*) of social media self-control failure (*b* = −0.23, *df* = 97, *t* = −2.71, *p* < .01). However, no significant prediction of general social media use on engagement was observed when the prediction was estimated at a higher level (+1 *SD*) of social media self-control failure (*b* = 0.04, *df* = 97, *t* = 0.50, *p* = .615). The main effect of general social media use and social media self-control failure was also found. Overall, students who used more social media in general or students who had a higher level of social media self-control failure reported a lower level of school engagement. Regarding burnout, neither an interaction between general social media use and social media self-control failure nor any main effect was found (Table 4).

**Table 4:** Moderation effects of general social media use on the relationship between social media self-control failure and the outcome measures.

Variables	Engagement				Burnout			
	Model 1		Model 2		Model 1		Model 2	
Control variables	*b*	*SE*	*b*	*SE*	*b*	*SE*	*b*	*SE*
Academic pressure	−0.17	0.10	−0.15	0.10	0.39***	0.07	0.34***	0.06
Self-control	0.39***	0.13	0.29**	0.13	−0.21*	0.08	−0.11	0.09
Depletion Sensitivity	−0.17	0.11	−0.15	0.10	0.12	0.07	0.13	0.07
Social media use intensity	0.16*	0.10	0.25**	0.11	−0.01	0.07	−0.05	0.07
Age	−0.04	0.04	0.02	0.04	−0.02	0.03	−0.04	0.03
Grade	0.16*	0.10	0.12	0.10	−0.03	0.07	0.00	0.07
Gender					0.06	0.10	0.05	0.10
General social media use (GSMU)			−0.11**	0.24			0.11	0.15
Social media self-control failure (SMSCF)			−0.13**	0.30			0.22	0.19
GSMU × SMSCF			0.16*	0.07			0.04	0.04
*F*	16.17***	12.91***			23.76***	19.53***		
*R^2^*	0.49	0.54			0.63	0.67		
Δ*R^2^*		0.05*			0.04*			

Coefficients were standardized.

* *p* < .05.

** *p* < .01.

*** *p* < .001.

## 4 Discussion

The study aimed to answer the question of whether high school students' social media use will predict more school engagement and less burnout for those who were more successful in controlling their social media use. Based on the study demands-resources model, we tested the hypothesis through an online survey among Chinese high school students. To our knowledge, this is the first study to examine the boundary condition of self-control in the relationship between social media use and student engagement and burnout. It provides empirical evidence regarding the positive aspect of social media use on high school students' school performance, which was relatively understudied in the existing literature.

### 4.1 The moderating role of social media use on the relationship between social media self-control failure and engagement

As expected, for high school students who were more successful in controlling social media use in goal-conflict situations, their general social media use (rather than social media use intensity) predicted more engagement. This result supported our assumption that more controlled social media use will provide opportunities and resources for high school students to engage in academic learning, which, as a result, promote student engagement. The results also supported the study demands-resources model (20) and added to the literature regarding the role of social media use in high school students' engagement.

The results may also support the idea of motivational interference. It was argued that when students are confronted with a motivational conflict between their learning goals and alternative activities they find attractive, their self-control will be impaired by the interference of these competing activities (44, 45). Our findings further indicated that such motivational conflict exists between students' important goals, including academic goals, and their use of social media. The use of social media platforms that elicit strong emotional rewards, such as online chatting and watching short videos, can disrupt students' motivation to learn. Nevertheless, students who were more successful in managing this motivational conflict were better able to resist the distractions of social media and maintain their focus on learning goals. This ability increases the potential for using social media as a tool to enhance engagement rather than leading to burnout.

It should be noted that we planned to use the Social Media Use Intensity scale to represent students' social media use because this measure delves into more specific activities on social media (e.g., social contact, entertainment, news reading, gaming) (53). However, no moderation or main effect was observed, whereas in exploratory analysis, general social media use (i.e., the time spent on and frequency of visits to social media) showed both moderation and main effect in predicting engagement. The reason could be that using a total score of the social media use intensity scale as an index of social media use may counteract the differences among various online activities in different persons across sample^1^. Future studies could separately examine the usage of different activities on social media when examining its effect on engagement.

Moreover, in the present study, the most popular social media platforms of high school students were WeChat (95%), QQ (89%) and TikTok (79%, Chinese name: Douyin). A prior study showed that TikTok was particularly attractive to Chinese students and was found to generate detrimental effects on adolescents' mental health such as depression and memory loss (59). The present study did not focus on specific social media platform in the analysis. However, it could be that for some social media platforms, the moderation effect of self-control on the association between social media usage and the outcome variables was more significant. Thus, future studies could further replicate the study regarding different social media platforms to better understand the impact of social media use on students' engagement and burnout. The results may contribute to establishing better guidance regarding high school students' social media use, especially for senior grade students and return students. We found that when students were able to regulate the conflict between social media use and study goals, more usage improved engagement at school. This may suggest that total abstinence from social media use may not be applicable for all students. A previous study showed that users who were abstinent from social media had a decline in wellbeing (60). Additionally, when people were not allowed to use social media for one day, they showed no difference in wellbeing compared to normal days (61). Our results add to the literature by showing that for high school students, the controllable use of social media can promote school engagement by facilitating connections to study resources. Thus, educators may consider encouraging controlled social media use in high school to increase engagement.

The findings from the research also indicate the importance of teachers finding effective ways to integrate social media into the classroom to enhance the study engagement of senior high school students. It is suggested that teachers focus on increasing students' ability to control their social media use. For example, by improving their digital self-efficacy, which refers to their confidence and ability to navigate and use digital platforms effectively (62), students will be better equipped to manage their social media usage and utilize it as a valuable tool for academic purposes, rather than a distraction. By becoming more proficient in using social media for educational purposes, students can establish a balance between recreational and educational activities on these platforms (62).

### 4.2 The moderating role of social media use on the relationship between social media self-control failure and burnout

Unexpectedly, high school students' social media use did not predict burnout regardless of how successful they were able to control their social media use. No main effect of the predictors was observed when considering the control variables. We hypothesized that high school students who were unable to control their social media use would be exposed more in goal-conflict situations between social media use and study goals. They might have a higher cognitive load in dealing with conflict dilemmas and suffer more from the time pressure and stress of unfinished tasks, which results in a higher level of burnout.

One of the reasons that we did not find support for the hypothesis could be that the association between social media use and burnout might be complex (63). Uncontrolled social media use could be an outcome rather than a predictor of burnout. It could also show a reinforcing influential pattern with burnout in the long run, which could not be observed with the current cross-sectional design. In a previous study, student burnout was found to predict sleep disturbance caused by social media (63). Another study demonstrated the mutual influences of students' excessive internet use and their burnout during a period of time (64). The findings suggest that burnout might be the predictor, or both predictor and outcome of uncontrolled social media use. Moreover, it was argued that burnout reflects a relatively long-term change in emotional state, which could fluctuate during a period of time (25). However, in the present study, we only examined the relationship between social media use and burnout at one time point. Thus, future research could use a longitudinal design to further test the association between students’ social media use and subsequent burnout or to explore their reciprocal relationships over time.

Another reason might be that we used a self-report measure to assess how successful students believed that they were able to control social media when it conflicts with other goals (i.e., Social Media Self-Control Failure scale). However, the explicit belief might not reflect people's actual behavioral tendency in real goal-conflict situations. It could be that social media use predicts less burnout only for students who not only had more successful belief in their self-control ability but also showed less implicit behavioral tendency toward social media. In support of this idea, a previous study found an inconsistency between social media users’ explicit self-report measure and implicit behavioral tendency toward social media in predicting excessive social media use (16). The study showed that an implicit attitude toward social media significantly predicted excessive social media, regardless of how successful people believed they could control social media use (16). Likewise, using an in-class design, another study found that whether or not a student believed that he or she could successfully control multitasking behaviors on computers, their social media usage constantly showed a negative effect on task performance (65). In the present study, a lack of implicit measurement regarding students' behavioral tendency toward social media limited the measurement to distinguish more successful (vs. less successful) social media users more precisely. Future studies could combine both explicit and implicit measures to study the impact of social media use on students' burnout.

Finally, we found that both general social media use and social media use intensity failed to predict burnout regardless of students' self-control level. The results were obtained while controlling for the effects of general self-control ability, the depletion sensitivity of self-control and perceived academic pressure. According to pairwise correlations, these control variables were highly correlated with burnout (all *r* > 0.5). Meanwhile, burnout had a nonsignificant correlation with social media use intensity or only a small correlation with general social media use compared to its high correlation with social media self-control failure. This reveals that social media self-control and its related constructs (i.e., general self-control ability and depletion sensitivity) might be more significant than social media *per se* in explaining high school students' burnout. This idea was supported by a prior study that only found a significant correlation between social media self-control failure (rather than general social media use) and affective wellbeing [e.g., (37)]. It could be that other alternative models might better explain the pathways from social media use to burnout, which is worth further exploration.

### 4.3 Limitations

Several limitations of the study should be noted. First, as an exploratory step, we collected a relatively small sample size to examine the effect of social media use on students' engagement and burnout. This is because the authors have conducted a prior study with similar variables based on a sample of 249 Chinese adolescents between 12 and 17 years old. The study demonstrated that students' academic performance was negatively associated with social media self-control failure, and the correlation index was significantly higher than its association with social media use. To further clarify the influential paths between social media use, social media self-control failure, and adolescents' school performance, we have conducted the current study based on the demands-resources model. In line with the pre-registration, we have also conducted the power analysis with a median effect size based on the correlation index of the prior study. However, the sample was mostly (88%) high school *senior* students (i.e., 3rd-grade students and returning students). Although this sample characteristic provides a novel perspective to look at students with particularly high study demands, it may also reduce the representativeness of high school students. Thus, any conclusion regarding the generalization of the results should be drawn with caution. Second, we used a cross-sectional design to study the moderation effect of self-control over social media use. The results only provide correlational results of the main variables. Caution should be taken when claiming any causal relationship between social media use and student engagement and burnout. Third, many Chinese high school students were resident students. Their social media use and school regulations regarding smartphone use might be different from regular students (e.g., resident students could be totally banned from smartphones during weekdays). Although we separately measured students' general social media use during weekends and weekends, the different statuses of residence may also affect the results, which should be considered in future studies. Last, given privacy concerns, we did not collect information regarding students' socioeconomic status (e.g., household income, educational level of parents). However, the potential impact of socioeconomic status on the results should also be take it into account.

## 5 Conclusion

Based on the demands-resources model, this study found that students who were more successful in controlling their social media use were more engaged in study. This implies that students who are able to effectively manage their time and restrict their social media usage when it disturbs other important goals are more likely to dedicate themselves to their schoolwork. However, the study did not find any significant correlation between social media use and burnout, regardless of how well a student could control their social media use. These findings suggest that acquiring successful self-control over social media usage could potentially enhance a student's engagement and commitment towards their studies. This, in turn, may significantly contribute to their academic achievements, especially for high school senior students. Replication of the analysis is necessary to further examine the generalization of the current findings in students with different grades and educational statuses. Also, the following studies are needed to establish a causal relationship between social media use, social media self-control, and high school students' engagement and burnout.

## Data Availability

The original contributions presented in the study are included in the article/Supplementary Material, further inquiries can be directed to the corresponding author.

## Footnotes

^1^The original scale distinguished two groups of activities that relate to the entertainment function and social function of social media. When separately examining their prediction on engagement and burnout and the moderation effect of social media self-control failure, no conclusion was changed.
